# The Removal of Tophi

**Published:** 1908-11-28

**Authors:** T. Churton

**Affiliations:** Senior Physician Leeds General Infirmary. Leeds


### EDITOR'S LETTER-BOX.

### THE REMOVAL OF TOPHI.

*To the Editor of* THE HOSPITAL.

SIR,—On September 28 last, a man, aged forty, who had suffered for two years from disabling "chalk-stones" or tophi, some of them very large, on his hands and elbows, was admitted under my care into the Leeds General Infirmary. It occurred to me that if the acicular crystals could be thoroughly removed and normal connective tissue and endothelial cells left, no further deposition of the crystals need follow, inasmuch as the patient was not now actually gouty, and the masses were, in effect, mere tumours. I therefore, on October 17, requested the resident surgical officer (Mr. R. L. Braithwaite) to incise and scrape out a small one, and when this was successfully done—the wound healing in five days—three larger masses, one of them on the back of the hand, the size of half a Tangerine orange, were similarly treated. We propose to evacuate those which remain and to report the success or the failure in due time. Meanwhile, though priority or "independence" is a very little, and very elusive, thing, it may be not improper to lay a small claim to our having entertained the idea of removal independently of, and perhaps contemporaneously with, Professor O. Laurent, whose case is mentioned in THE HOSPITAL of November 21, page 188, column 2, paragraph 1.

Yours faithfully,
T. CHURTON, M.D.,
Senior Physician Leeds General Infirmary.

Leeds: November 23, 1908.

A SPECIAL matinée was given on November 18 at the Court Theatre by Mr. D. Brandon in aid of the funds of the Hospital for Sick Children, Great Ormond Street, and proved very successful. The plays, which were three in number, were produced under the direction of Mr. Patrick Kirwan, and a large audience received them with every mark of appreciation.

